# Genetic Research and Women’s Heart Disease: a Primer

**DOI:** 10.1007/s11883-016-0618-x

**Published:** 2016-10-10

**Authors:** Maryam Kavousi, Lawrence F. Bielak, Patricia A. Peyser

**Affiliations:** 1 Department of Epidemiology, Erasmus University Medical Center, Rotterdam, P.O. Box 2040, 3000 CA Rotterdam, The Netherlands; 2 Department of Epidemiology, School of Public Health, University of Michigan, 1415 Washington Heights, Ann Arbor, MI 48109-2029 USA

**Keywords:** Cardiovascular disease, Coronary heart disease, Atherosclerosis, Women, Genes, Sex-differences

## Abstract

**Purpose of Review:**

This review provides a brief synopsis of sexual dimorphism in atherosclerosis with an emphasis on genetic studies aimed to better understand the atherosclerotic process and clinical outcomes in women. Such studies are warranted because development of atherosclerosis, impact of several traditional risk factors, and burden of coronary heart disease (CHD) differ between women and men.

**Recent Findings:**

While most candidate gene studies pool women and men and adjust for sex, some sex-specific studies provide evidence of association between candidate genes and prevalent and incident CHD in women. So far, most genome-wide association studies (GWAS) also failed to consider sex-specific associations. The few GWAS focused on women tended to have small sample sizes and insufficient power to reject the null hypothesis of no association even if associations exist.

**Summary:**

Few studies consider that sex can modify the effect of gene variants on CHD. Sufficiently large-scale genetic studies in women of different race/ethnic groups, taking into account possible gene-gene and gene-environment interactions as well as hormone-mediated epigenetic mechanisms, are needed. Using the same disease definition for women and men might not be appropriate. Accurate phenotyping and inclusion of relevant outcomes in women, together with targeting the entire spectrum of atherosclerosis, could help address the contribution of genes to sexual dimorphism in atherosclerosis. Discovered genetic loci should be taken forward for replication and functional studies to elucidate the plausible underlying biological mechanisms. A better understanding of the etiology of atherosclerosis in women would facilitate future prevention efforts and interventions.

## Introduction

Cardiovascular disease (CVD) remains the leading cause of mortality among women and men [1, 2]. Although overall CVD mortality rates have declined, the annual mortality rate for women remains higher than men [3]. Greater life expectancy for women, together with improvements in primary and secondary prevention of CVD, will lead to a larger proportion of women living with CVD [3]. Substantial sex differences in the burden of different manifestations of CVD, including coronary heart disease (CHD), stroke, heart failure, and peripheral artery disease, are widely recognized [4–7]. Despite the excess CHD incidence and prevalence in men compared to women, CHD remains the leading contributor to CVD morbidity and mortality among both women and men [1, 2].

In spite of statistics that show CHD develops on average 7–10 years later in women compared with men, adverse trends in many risk factors among women are of growing concern [3, 8]. Additionally, while the decrease in CHD mortality among women is well documented, the decline still lags behind that of men, with an alarming tendency towards an increase mortality rate among younger women [9, 10•]. Recent data report substantial declines in sudden cardiac death in men while no changes are observed among women [11].

We provide a review of sex differences in atherosclerosis, the contribution of genetic research to explaining sex differences in atherosclerosis, possible sex hormone-mediated epigenetic mechanisms, and use of subclinical measures of atherosclerosis for genetic studies. Next, we discuss challenges in accounting for sex differences in genetic studies, importance of the proper definition of outcomes, the need to include multiple race/ethnic groups in genetic studies of atherosclerosis in women, and how this genetic information can contribute to efforts in precision medicine for CHD in women. We conclude with directions for future research, limitations of this review, and conclusions. Here, sex refers to biological differences between women and men (i.e., anatomical and physiological differences, genetic differences in the X and Y chromosomes, and levels and types of hormones). Gender is “the socially constructed characteristics of women and men—such as norms, roles and relationships of and between groups of women and men. It varies from society to society and can be changed” (http://www.who.int/gender-equity-rights/understanding/gender-definition/en/).

### Sex Differences in Atherosclerosis

CHD is mainly characterized by atherosclerosis in the epicardial coronary arteries. Atherosclerosis is a systemic progressive pathologic condition involving atherosclerotic plaque formation typified by accumulation of cholesterol, infiltration of macrophages, proliferation of smooth muscle cells, accumulation of connective tissue components, and formation of thrombus [12]. Atherosclerosis is considered a complex trait involving multiple genes and their interactions with behavioral and environmental factors. Complex traits do not follow predictable patterns of inheritance. Although women and men share many similarities in core processes underlying atherosclerosis, recent evidence points towards some inherent differences in the development of the disease. Sex differences in the association of traditional risk factors with CHD are established. In particular, smoking and diabetes seem to be stronger risk factors in women [13, 14]. Sex differences, however, cannot be entirely explained by the differential distribution of traditional risk factors between women and men. Furthermore, women tend to show a more “diffuse atherosclerosis” pattern, as opposed to discrete “focal atherosclerosis” that obstructs the lumen [15].Compared to men, women tend to have a higher prevalence of microvascular dysfunction [15, 16]. Plaque characteristics relative to calcification and lipid accumulation may also differ between women and men, and transitions toward vulnerable plaques seem to be slower in females [15]. Sex differences in inflammatory, coagulation, and thrombotic pathways may contribute to this sexual dimorphism [15–17]. The exact pathways associated with atherosclerosis in women and men, however, remain to be elucidated.

### Contribution of Genetic Research to Explaining Sex Differences in Atherosclerosis

The heritability of CHD is approximately 40 % [18]. Several studies have shown that a family history of CHD, especially when disease occurs before age 60 years, is more important for women than men [19]. Understanding the genetic basis of sexual dimorphism in atherogenesis may provide novel additions to existing knowledge.

### I. Candidate Genes for Atherosclerosis

A gene whose function or location indicates it is likely to be responsible for a particular disease or trait level is referred to as a candidate gene. Detailed information about specific genes can be found at http://www.ncbi.nlm.nih.gov/gene. Most candidate gene studies pool women and men and use sex as a covariate for adjustment. In the selected studies described below, men and women were considered separately. While some studies found associations in men, but not women, we only report those where an association only occurred in women.

#### Apolipoprotein E

One of the most studied candidate genes for CHD is apolipoprotein E (APOE) [20]. APOE produces a protein involved in metabolism of cholesterol and triglycerides by binding to receptors in the liver to promote clearance of chylomicrons and very low-density lipoproteins from circulating blood [20]. The major alleles of APOE are ε2, ε3, and ε4. The most common genotypes are ε3 homozygotes, ε3/ε4 heterozygotes, and ε2/ε3 heterozygotes in most populations. ε2/ε3 heterozygotes have higher high-density lipoprotein cholesterol (HDL-C) levels while ε3/ε4 heterozygotes have higher low-density lipoprotein cholesterol (LDL-C) levels compared to ε3 homozygotes [20]. ε3/ε4 heterozygotes have higher risk of CHD compared to ε3 homozygotes and ε2/ε3 heterozygotes, while there is no significant difference in CHD risk between ε3 homozygotes and ε2/ε3 heterozygotes [20].

The Framingham Offspring Study, one of the earliest studies to demonstrate a gender-specific association between ε4 and prevalent CHD, found a significant positive association in women, but not men, after adjusting for age and traditional CHD risk factors [21]. No protective effect of ε2 was found in either women or men in this study of 1034 men and 916 women. More recently, a large prospective study of 10,035 men and 12,134 women from the Norfolk, England, arm of the European Prospective Investigation into Cancer and Nutrition study (EPIC Norfolk study), reported no association between CHD risk and *APOE* in either men or women after adjustment for multiple traditional risk factors [22]. While both the Framingham Study and the EPIC Norfolk study adjusted for LDL-C and HDL-C levels, the Framingham Study assessed prevalence of CHD while the EPIC Norfolk study assessed incident CHD over 11 years. Finally, the EPIC Norfolk study also included alcohol use and physical activity as risk factors. The large, prospective EPIC Norfolk study supports a limited role for *APOE* in risk of CHD for either sex after accounting for many CHD risk factors [22]. Importantly, these studies suggest that genes for prevalent CHD may differ from genes for incident CHD.

#### Selected Other Candidate Genes

In two large Finnish cohorts, 46 candidate genes were studied for association with CHD [23]. No variants in any candidate gene were associated with incident CHD in men. Variants in three genes, however, showed an association with incident CHD in women: upstream stimulatory factor 1 (*UFS1*), coagulation factor XIII A (*F13A1*), and carboxypeptidase B2 (*CPB2*). *UFS1*, which was also associated with prevalent CHD in women, is a ubiquitously expressed transcription factor that regulates several genes of glucose and lipid metabolism. *F13A1* is involved in the blood coagulation cascade while *CPB2* is involved in fibrinolysis. Additionally, mutations in either of two polymorphisms in the hemochromatosis (*HFE*) gene in the Rotterdam Study were significantly associated with incident CHD in women, but not men [24]. *HFE* regulates circulatory iron uptake. All the candidate gene studies described above included only individuals of European ancestry.

The candidate gene approach has been criticized primarily because many results could not be replicated and this approach fails to include all possible causative genes and polymorphisms [25]. These criticisms spurred new approaches such as genome-wide association studies (GWAS) to identify genes for complex diseases and traits

### II. Genome-Wide Association Studies

GWAS typically consider millions of single-nucleotide polymorphisms (SNPs) (or other genetic variants such as copy number polymorphisms). Genetic variants are measured with microarrays, and the measures from the microarrays are often combined with publically available data, such as the 1000 Genomes Project, to impute additional genotypes that were not directly measured. The 1000 Genomes Project has provided genomic sequence data on more than 2500 individuals from 26 globally diverse populations [26].

In contrast to candidate gene studies, there are no specific a priori hypotheses in GWAS with the exception of replication of already identified genes. These studies typically meta-analyze results from multiple cohorts to increase power. There is usually a discovery phase that includes the initial cohorts and then a replication phase with additional new cohorts. Cohorts from the discovery and replication phases may be meta-analyzed together. Depending on the number of SNPs considered, the *p* value is set to meet a Bonferroni correction. Many GWAS of traditional CHD risk factors explore whether any identified genes are also associated with clinical outcomes. A catalogue, continually updated, of GWAS findings is available [27••].

The first GWAS papers on CHD were published in 2007 [28–32]. The studies pooled women and men of European ancestry and adjusted for sex as a covariate. The most consistent finding in these studies was an association with variants on the short arm of chromosome 9 (9p21).

Recent GWAS have included more genetic variants and larger sample sizes. The most recent GWAS included 60,801 CHD cases and 123,504 controls from 48 different cohorts [33••]. While most of the cases and controls were of European ancestry, some were of other ancestries. The study interrogated 9.4 million variants across the genome. Ten new loci were identified bringing the total number of loci associated with CHD to 58 [33••, 34••].

Sex-specific associations for CHD were conducted in the Wellcome Trust Case Control Consortium. In women, there were 399 cases for CHD and 1492 controls while in men there were 1527 cases and 1446 controls. Variants considered were selected from results of other GWAS for CHD. No variants were significant in women (or in men) [35]. Power was low in this study.

In a recent sex-stratified study, an SNP in *SCARB1*, a plasma membrane receptor for HDL, was associated with angiographic CHD in women, but not men [36•]. A GWAS of betaine levels, a novel risk factor for atherosclerosis, found an association with variation in a SNP in carbamoyl-phosphate synthase 1 (*CPS1*) [37••]. *CPS1* encodes a mitochondrial enzyme that catalyzes the first committed reaction and rate-limiting step in the urea cycle. This SNP was weakly associated (*p* = 0.01) with CHD in approximately 54,000 individuals from the CARDIoGRAM Consortium [38]. The SNP was not associated with CHD in men; however, it was significantly associated with CHD in women [37••].

All CHD GWAS so far focused exclusively on the autosomes even though the X chromosome is included on all the current microarrays [39]. The X chromosome contains 1973 known genes. Even with the challenges of including the X chromosome in GWAS because women have two X chromosomes while men have one and because of X-inactivation, this chromosome may provide important information regarding differences in atherosclerosis and its risk factors between women and men. The one exception is a recent GWAS of nonobstructive coronary artery disease in women that included 52,371 variants important in metabolic traits and CVD [40, 41]. Ninety variants were on the X chromosome. The 332 European ancestry cases came from a cohort of women with chest pain and/or suspected myocardial ischemia with <50 % stenosis in any coronary artery on angiography. The 1003 European ancestry controls came from a cohort of women without known CHD. While there were no associations with any SNPs on the X chromosome, SNPs at two autosomal genes showed association at nominal significance levels. In a candidate gene study of *5HTR2C* on the X chromosome, men who had one copy of the high-risk allele and women with two copies of the high-risk allele were at significantly increased risk for death or nonfatal myocardial infarction [42].

### Sex Hormones and Epigenetics

CHD in women tends to manifest during and after the menopausal transition, indicating that sex hormones play a critical role in disease development. The major impact of sex hormones on atherosclerosis, either directly affecting the function of the heart and vessels or indirectly through other CHD risk factors, has long been investigated [43]. A recent understanding of the interaction of genes with the environment has revealed the importance of sex hormones on pathogenesis of atherosclerosis through epigenetic mechanisms.

Epigenetics refers to heritable changes in gene activity and expression that do not entail an alteration in DNA sequence. In other words, epigenetics involves the molecular pathways that modulate the expression of a genotype into a particular phenotype [44]. Epigenetic modifications often investigated include DNA methylation, histone variants, and histone modifications as well as nucleosome positioning [45]. Epigenetic mechanisms are reversible and can be modulated by environmental factors [46]. Thus, epigenetics has emerged as a promising tool to address knowledge gaps in atherosclerosis.

CHD risk factors, such as nutrition, smoking, pollution, stress, and the circadian rhythm, have been associated with epigenetic modifications [47•]. Additionally, sex hormones are uniquely poised to exert epigenetic effects through hormone-induced DNA methylation and histone modification at specific gene regulatory regions [48]. Any evidence regarding a sex-specific association of a particular genetic locus with atherosclerosis could indicate underlying epigenetic mechanisms mediated through sex hormones.

### Subclinical Measures of Atherosclerosis

The distribution of atherosclerosis in different vascular beds is variable and sex- and race/ethnicity-specific [49–52]. Differences in atherosclerosis between men and women have been suggested to be larger in the coronary arteries than in other vascular beds [49]. Calcification in the coronary arteries is a surrogate marker of overall plaque burden and is considered the hallmark of atherosclerosis. While women have overall lower presence and quantity of coronary artery calcification (CAC) than men, CAC carries a higher mortality risk for women [53]. The quantity of CAC is heritable [54]. So far, none of the GWAS for CAC have considered men and women separately [55•, 56]. The GWAS for CAC in those of European ancestry identified associations with SNPs in the 9p21 region and in *PHACTR1* on chromosome 6 that replicated for myocardial infarction [55•]. A GWAS for CAC in those of African ancestry failed to identify any genome-wide significant associations [56].

Few candidate gene studies have evaluated genetic associations for CAC in men and women separately. A polymorphism in E-selectin, a gene involved in cellular adhesion, was associated with presence of CAC only in women age 50 years or younger after adjustment for CHD risk factors. There was no association with CAC presence in men of any age or in women over age 50 after adjustment for CHD risk factors [57]. A promoter polymorphism of leukotriene C4 synthase (*LTC4S*), the rate-limiting enzyme in the production of the potent proinflammatory cysteinyl leukotriene metabolites of arachidonic acid, was studied in women ages 29–43 years and men ages 29–37 years [58]. Risk for having CAC was significantly associated with this polymorphism in women, but not men.

Increased common carotid intima-media thickness (CIMT) is considered a marker of early atherosclerosis. Sex differences in CIMT seem to be pertinent only in younger and middle-aged individuals, becoming progressively irrelevant at older age [59]. In a longitudinal study of CIMT, progression of CIMT was associated with parental history of stroke especially among young women [60]. CIMT is heritable [61]. A GWAS in those of European ancestry identified variants near *ZHX2*, *ACPOC1*, and *PINX1* associated with CIMT and did not consider women and men separately [62•]. A recent GWAS in a study that included individuals from multiple race/ethnic groups identified 14 genes with evidence for an association with CIMT [63•]. An SNP-by-sex interaction was found for a SNP in *LEKR1* and an SNP in *GALNT10*. Both of these loci have been associated with adiposity and weight control [63•]. Similar to the association with CAC described above, *LTC4S* was associated with CIMT in women but not in men [58]. Others have identified a locus on chromosome 16 that is associated with CIMT, which contains the *BCAR1*, *CFDP1*, and *TMEM170A* genes [64]. More recently, Boardman-Pretty and colleagues [65•] studied the lead SNP in *BCAR1*, identified its function, and showed it to be associated with progression of CIMT in women, but not men.

Endothelial function, a measure of physiological functions of the vascular endothelium, is a marker representing the effects of CHD risk factors on the arterial wall [66, 67]. Functional and structural damages to the arterial wall precede and accompany atherosclerosis process and its associated obstructive and thrombotic events [66]. Sex has been suggested as an independent factor contributing to endothelial dysfunction, and the effect of cardiovascular risk factors on endothelium-dependent dilation has been shown to be sex-specific [68, 69]. Endothelial function is heritable [70]. An early GWAS did not find any genome-wide significant associations but found some suggestive associations for endothelial function [71]. A recent study investigated the association between almost 1300 SNPs previously associated with vasoreactivity, angiogenesis, inflammation, artery calcification, atherosclerotic risk factors, insulin resistance, hormone levels, blood coagulability, or CHD with coronary endothelial dysfunction [72]. SNPs in *LPA*, *MYBPH*, *ADORA3*, and *PON1* were significantly associated in the 426 women, but not in the 217 men.

### Accounting for Sex Differences in Genetic Studies: the Challenge

Analyzing sex differences in genetic associations is complicated, particularly for a disease such as CHD where the typical age of onset is gender-dependent. Sufficiently powered studies are an essential requirement in both candidate gene and GWAS studies. Lack of adequate statistical power has hampered meaningful comparisons between women and men in most of the numerous published GWAS for CHD so far. The sex-specific GWAS in the Wellcome Trust Case Control Consortium [35] and the study of nonobstructive coronary disease in women [40] illustrate the challenges of smaller samples and the need for large-scale studies in women.

Importantly, when women and men are pooled in a study, the analysis of any variant, but especially rare genetic variants, might be prone to bias resulting from the disproportionate blend of women and men in case and control samples. Other genetic or environmental factors can modify or mediate the effect of a particular genetic variant to increase or decrease the risk for CHD in women. For example, a study with a large sample of men and women found an interaction between smoking and *APOE* on CHD risk [73]. Women, but not men, had an increased smoking-related risk in ε4 allele carriers. Differences between men and women can result from the joint effects of genetic variants with other biological and/or environmental variables.

While investigating sex differences in genetic studies of atherosclerosis are of paramount importance, researchers should be aware not to overstate sex differences in their studies, as spurious claims of sex differences in genetic studies might be asserted in the absence of sufficient data, proper data analysis, or good internal and external validity [74•]. Bias against reporting negative or null results can lead to more publications conveying findings of sex differences compared to studies with no sex differences appearing in the literature [75].

Since the sex-specific differences may be larger for causal variants than for their linked markers that might be studied, systematic assessment should be aimed at targeting the true causal variants associated with atherosclerosis [35]. Candidate gene studies should be based on a priori, clearly defined hypotheses. Any claim of sex-specific genetic association should be accompanied by appropriate examination of subgroup comparisons or interaction tests. Likewise, gene-gene and gene-environment interactions should be interpreted with caution and viewed in the context of additional prior/external evidence. The identified interactions should be interpreted as hypothesis-generating, followed by further replication in other studies as well as meta-analyses [76, 77].

### Proper Definition of Outcome in Sex-specific Genetic Studies of Atherosclerosis

In studies of complex disease, defining a trait with insufficient specificity, i.e., trait heterogeneity, is viewed as a confounding factor [78]. The pathophysiology of CHD involves more severe structural and functional abnormalities in epicardial coronary arteries in men and more microvascular coronary dysfunction in women [79]. Thus, using the same definition for the CHD phenotype for both women and men might not be appropriate and could lead to differential misclassification of cases and controls between women and men. For example, in the most recent GWAS for CHD, case status was defined by an inclusive CAD diagnosis including myocardial infarction, acute coronary syndrome, chronic stable angina, or coronary stenosis of >50 % [33••]. Accordingly, the term ischemic heart disease (IHD) is more appropriate for women than CHD. Abnormal coronary reactivity, microvascular dysfunction, and plaque erosion/distal microembolization contribute to a female-specific IHD pathophysiology. Using IHD, rather than obstructive CHD, covers the whole spectrum of the disease in women and could allow further identification of genes in studies focusing on women [15].

Another issue is whether studies have included prevalent or incident CHD cases. A recent GWAS was the first to investigate incident cases [80••]. Importantly, a new gene was identified that was not identified in prior GWAS of prevalent cases while the 9p21 region and other genes identified in studies with prevalent cases were only marginally associated or not associated with incident disease. One explanation is that SNPs in the 9p21 region are associated with increased risk as well as survival after onset of CHD.

### Racial Disparities in Genetics of Coronary Heart Disease in Women

The majority of genetic studies include only those of European ancestry. The differences among the various race/ethnic groups in CHD morbidity and mortality are well documented [2]. The importance of sex and race/ethnicity in genetic studies of CVD has been recognized [81]. One GWAS of incident CHD was conducted in African Americans [82]. The discovery cohort included both women and men, but the replication cohort included only women. One SNP near *PFTAIRE-1*, which is involved in cell proliferation, was genome-wide significant and replicated. The region was not implicated in the incident CHD GWAS that pooled men and women [80••].

A recent study was designed to understand how to facilitate inclusion of minority individuals in genetic studies [83]. Starting with a large random sample from a community, they performed telephone screening interviews, determined eligibility, and then recruited participants for a genetic study on dependence on cigarettes and nicotine. In zip codes with a high proportion of African Americans, compared to those with very low proportions of African Americans, there was a significantly higher proportion of individuals with incorrect telephone numbers or addresses but a lower proportion of individuals who did not answer the telephone or refused the interview. Importantly, a significantly higher proportion of eligible African Americans participated in the genetic study compared to eligible European Americans (71 % versus 54 %). Results suggest that increasing the number of African Americans in genetic studies and registries may be achieved by increasing efforts to locate and contact them. This study did not address some other issues regarding possible lack of participation of minorities in research studies because of mistrust or limited access to health care.

### Genetics of Women’s Heart Disease: the Biological Basis for Precision Medicine

Contrary to the traditional approach of “one size fits all,” precision medicine aims to tailor disease prevention, treatment, and prognosis regimens [84•]. Sex-specific CHD research will lead to a new understanding of the pathophysiology of CHD in women, allowing for a comprehensive disease definition and classification. Taking into account individual variability in the role of genetic, environmental, and social factors as well as their interaction in the pathophysiology of atherosclerosis would serve to implement effective preventive or therapeutic interventions. As gene therapy becomes a promising new addition to conventional therapies, the sex-specific as well as the race/ethnic-specific genetic basis of the atherosclerosis assumes more significance.

### Directions for Future Research

CHD is increasingly recognized as a pathophysiological continuum [85], emphasizing the notion that intervention at any point along this path can modify disease progression. Disease progression does not occur as a sequence of discrete, tandem incidents but instead the phases of disease progression overlap. Therefore, sex-specific studies across the entire spectrum of atherosclerosis are needed. Studying intermediate phenotypes in the continuum, including CAC, CIMT, or endothelial function, may help disentangle sex differences in disease susceptibility and progression. Moreover, focusing on quantitative atherosclerosis markers might overcome some of the limitations regarding the heterogeneity in discrete CHD event ascertainment for women and men. In GWAS, the imprecision involved in measurement of quantitative phenotypes may not have large systematic effects on location of significant associations, but assessing repeatability of the phenotype is recommended [86].

The vast majority of identified SNPs do not result in protein changes. Instead, they could be, for example, long noncoding RNA, microRNA (small noncoding RNA that functions in RNA silencing and post-transcriptional regulation), or some transcribed or regulatory element. Thus, understanding the function of the identified SNPs is crucial for understanding their role in the disease process [87••]. One approach is to use available databases such as Encyclopedia of DNA elements (ENCODE) to identify the function of specific SNPs. Another is to utilize the data collected by the Genotype-Tissue Expression (GTEx) project, which provides a database and associated tissue bank to study the relationship between genetic variation and gene expression in human tissues [88]. A complete list of available databases is updated each year [89•]. Another approach is to study the function of a specific SNP and genomic region. For example, Yang and colleagues [90••] focused on an SNP in the *COL4A2* gene that has been shown to be associated with CHD in GWAS. They studied the functional effect of this variant in primary cultures of vascular smooth cells and endothelial cells from individuals with different genotypes at this SNP. They found differences in gene expression levels of *COL4A2* and *COL4A1* (*COL4A1* and *COL4A2* reside next to each other and share common transcriptional regulatory sequences). Additional immunohistochemical and histological studies of *ex vivo* atherosclerotic coronary arteries identified plaque differences dependent on genotype. Together, these studies, as well as their research in patients with angiographically documented disease, provide a mechanistic explanation for the association between the genetic variants and CHD [90••]. One recent study used reporter gene assays, computational predictions, and epigenomic marks to assess activity of an enhancer region active in multiple human tissues [91] while another study used integrative genomic, epigenomic, and transcriptomic profiling of perturbed human coronary artery smooth muscle cells and tissues to begin to identify causal regulatory variation and mechanisms responsible for CHD associations [92•].

More recent studies are now examining exomes [93, 94••]. In addition, there are large-scale whole-genome sequencing projects in progress. The National Heart Lung and Blood Institute at the National Institutes of Health in the USA is sequencing more than 62,000 individuals from more than 30 studies, many with measures of CHD and risk factors through the Trans-Omics for Precision Medicine (TOPMed) Program (http://www.nhlbi.nih.gov/research/resources/nhlbi-precision-medicine-initiative/topmed). Hopefully, these various approaches will result in large samples of women for genomic studies of CHD.

### Limitations of Review

There are many articles in the literature on the genetics of CHD and its risk factors that could not be included. We did not consider other manifestations of CVD that are relevant for women such as stroke, heart failure, and peripheral artery disease. We included only candidate gene and GWAS studies based on microarray data and did not include any GWAS based on exome sequencing or whole genome sequencing data that are just becoming available.

## Conclusions

Sex-based differences in the heart, in vessel function, and in major manifestations of CVD have been recognized. The influence of sex on CHD is increasingly being explored at cellular, molecular, and genetic levels. Challenges and recommendations to account for sex differences in genetic studies of atherosclerosis are summarized in Table 1. So far, sex-specific genetic studies of atherosclerosis are sparse, mostly due to lack of power or lack of appreciation for sex differences. Sufficiently large hypothesis-free GWAS or candidate gene studies with a priori, clearly defined hypotheses are needed. Accurate phenotyping and inclusion of relevant outcomes in women, consideration of possible gene-gene and gene-environment interactions, and sex hormone-mediated epigenetic mechanisms are of importance. Finally, discovered genetic loci should be taken forward for replication and functional studies to elucidate plausible underlying biological mechanisms.

**Table 1:** Accounting for sex differences in genetic studies of atherosclerosis: challenges and recommendations

Challenges	Recommendations
Lack of meaningful comparisons between women and men due to inadequate statistical power	Design sufficiently powered studies
Bias resulting from disproportionate blend of women and men in case and control samples	Careful consideration of the proportion of women and men in case-control studies
Differential misclassification of cases and controls between women and men due to suboptimal accuracy in phenotyping (i.e., trait heterogeneity)	Define the trait with sufficient specificity taking into account sex differences in pathophysiology of atherosclerosis Conduct sex-specific studies across the entire spectrum of atherosclerosis and study the intermediate phenotypes Distinguish prevalent versus incident traits in genetic studies of atherosclerosis
Inconsistent results due to race/ethnic differences in CHD morbidity and mortality	Sufficient inclusion of individuals from different race/ethnic groups to investigate differences in genetic associations with atherosclerosis
Spurious findings due to the interaction of genes with other genetic and/or environmental factors	Perform a systematic genetic assessment targeting the true causal variants Apply proper data analysis techniques Perform appropriate examination of subgroup comparisons or interaction terms Consider possible sex hormone-mediated epigenetic mechanisms
Lack of comprehensive evidence supporting the biological relevance of findings	Assess the internal and external validity of findings Take forward the discovered genetic loci for replication Use available databases to identify function of genes and relationship between genetic variation and gene expression Undertake functional studies to understand the function of the identified genetic variants
